# Can ultrasound measures of intrinsic foot muscles and plantar soft tissues predict future diabetes-related foot disease? A systematic review

**DOI:** 10.1371/journal.pone.0199055

**Published:** 2018-06-15

**Authors:** Troy Morrison, Sara Jones, Ryan S. Causby, Kerry Thoirs

**Affiliations:** 1 School of Health Sciences, University of South Australia, Adelaide, South Australia; 2 International Centre for Allied Health Evidence (iCAHE), University of South Australia, Adelaide, South Australia; 3 Department of Rural Health, University of South Australia, Adelaide, South Australia; Weill Cornell Medicine-Qatar, QATAR

## Abstract

**Introduction:**

Diabetes mellitus (DM) is associated with hyperglycaemia and advanced glycosylation end-products. In the foot, the consequences of chronic or uncontrolled diabetes are micro and macrovascular disease, neuropathy, reduced joint mobility and structural and soft tissue changes that increase the risk of ulcer development and amputation. Diabetes foot assessment currently includes a comprehensive history, neurological and vascular assessments and examination focussed on dermatological and musculoskeletal abnormalities. Whilst these assessments are helpful for predicting ulceration risk, direct identifiers that enable early therapeutic intervention are lacking. The intention of this review was to ascertain if B-mode ultrasound could be clinically applied to identify structural change in the diabetic foot and be utilised as an early predictor of ulceration risk.

**Methods:**

Primary databases and grey literature sources were systematically searched. Selection criteria were that the study included a diabetic sample and used B-mode ultrasound to assess soft tissue structures of the foot (plantar skin, plantar fat pad or intrinsic muscles).

**Results:**

Fifteen studies were identified for inclusion (combined diabetic sample of 773). Ultrasound demonstrated reductions in tissue thickness in diabetics compared to non-diabetics under first (p = 0.01) and second (p = 0.03) metatarsal heads, but not the third (p = 0.24). Statistical heterogeneity was high for ultrasound thickness measures under metatarsal heads four/five (I^2^ 65%, 81%) and very high for plantar skin (I^2^ 98%), heel pad (I^2^ 76%) and intrinsic muscles (I^2^ 91%, 81%). Extensor digitorum brevis (EDB) ultrasound measures were significantly thinner in diabetics for all dimension measures compared to healthy controls except one study, which reported no significant differences in EDB thickness.

**Conclusions:**

No direct evidence was found to indicate B-mode ultrasound measures can predict soft tissue changes in the plantar foot in diabetes, although low level studies indicate ultrasound has the potential to identify structural change. Clinical, methodological and statistical heterogeneity limit result applicability. This review highlights the need for robust prospective longitudinal research to examine the predictive validity of this method.

## Introduction

Diabetes mellitus (DM) is a chronic metabolic disease that is predicted to impact over 640 million people by the year 2040 [1]. People with DM are at increased risk of developing diabetes-related foot complications with the risk of ulcer development reported at 15–25% [2, 3]. Up to 20% of diabetics with foot ulcers require amputation [4].

The plantar soft tissues of the foot protect, withstand load and absorb mechanical stresses, whilst the intrinsic foot muscles provide dynamic control and foot stability. These tissues can be compromised in chronic or uncontrolled DM which results in accelerated effects of glycosylation, leading to collagen and elastin degradation, limited joint mobility, micro and macrovascular angiopathies and peripheral nerve damage [5–7]. In the foot, the consequences of elastin and collagen degeneration are a loss of adipocyte chamber structure and altered tissue mechanical properties.

Soft tissue structural changes commonly occurring in the diabetic foot include fat pad atrophy, sub-metatarsal fat pad migration (mechanical displacement of the sub-metatarsal head fat pads) and intrinsic foot muscle atrophy [7–10]. Atrophy of intrinsic foot muscles leads to structural and gait changes which can alter foot biomechanics and subsequently increase plantar pressures, particularly under the metatarsal heads [11]. The combination of advanced glycosylation and fat pad atrophy or migration reduces the ability of the foot to adapt, resist load and absorb stresses associated with mechanical loading. Consequently, soft tissue breakdown is initiated with lower levels of stress [12], resulting in a foot that is more vulnerable to soft tissue damage during gait [9]. If peripheral neuropathy, impaired vascular perfusion and impaired immune response are added to this sequence, an ideal environment for ulceration and its complications is created [13], particularly in the absence of good clinical management.

There is no single tool that can predict with certainty, and at an early stage, those feet at direct risk of ulceration. Knowledge of sub-clinical soft tissue breakdown in this population would be useful and allow early clinical intervention to improve limb morbidity. Ultrasound imaging can efficiently and reliably assess the soft tissues of the foot [14, 15]. It is also safe, widely available clinically, and can assess tissues in real time. Therefore, ultrasound imaging could potentially be used in conjunction with existing clinical screening methods to prospectively assess the diabetic plantar foot in people at high risk of insensate injury and soft tissue breakdown, such as diabetic people with peripheral neuropathy [16].

The objective of this review was to investigate the evidence supporting the use of ultrasound imaging to evaluate structural changes in the soft tissues of the diabetic foot including the prediction of diabetes-related changes such as ulceration.

## Methods

Using PRISMA guidelines [17], a PICOS (participants, intervention, comparison, outcome and study) search strategy was developed (S1 Fig) to identify and review studies that used high resolution ultrasound to image the soft tissues of the plantar foot in people with DM.

Studies reporting measurements of the soft tissues of the plantar foot using two-dimensional (2D) B-mode ultrasound on human DM participants were eligible for inclusion. The primary outcome measures of interest were two-dimensional (2D) measures of plantar fat pad (or plantar soft tissue depth), plantar skin thickness or intrinsic foot musculature as determined by B-mode ultrasound. If reported, measurements of morphologic ultrasound tissue characteristics of the plantar soft tissues or intrinsic muscles were also included. Studies were excluded if they exclusively used any of the following ultrasound technologies: Doppler, three-dimensional, elastography, ultrasound palpation, M-mode or therapeutic ultrasound.

All aetiological and prognostic study designs (NHMRC [18–20]) were included with no restrictions on language, publication dates or participants including number of participant groups, DM type or duration, or participant age. Opinion papers, editorials and narrative reviews were excluded. Studies were not included if they were published in a journal which does not require ethical review committee approval in accordance with the World Medical Association Ethical Principles for Medical Research Involving Human Subjects (Declaration of Helsinki) [21].

Electronic database searches (PubMed, MEDLINE, EMBASE, CINAHL, AMED, ICONDA, Joanna Briggs Institute, Ovid Nursing Database, Cochrane Library) and manual searches (of reference lists) were performed from 9th of October to 14th of December 2016. Searches were last re-executed in December 2017 with no new eligible articles identified. The main search concepts (MeSH terms) were (i) diabetes mellitus, (ii) foot and (iii) ultrasonography (S1 Table). Search terms were expanded using synonyms.

Titles and abstracts were initially screened by TM using eligibility criteria. The full text of eligible articles were retrieved and independently reviewed by TM and KT. Full texts were also retrieved and reviewed if there was uncertainty for the eligibility. Disagreements were resolved by consensus.

Data extraction categories were developed by TM, KT and SJ and included study information (ultrasound outcome measures, ultrasound characteristics, study design, sampling, diabetes characteristics) and participant information (diabetes status, age, gender, BMI, foot measured). Data extraction was performed by TM and checked for accuracy by KT.

The quality of each report was independently assessed by TM and KT against a critical appraisal tool for quantitative studies developed by Law and Colleagues [22]. Disagreements were resolved by SJ. Risk of bias was assessed using criteria described within the Cochrane Handbook [23, 24] (S2 and S3 Figs). Funnel plots were generated to identify publication bias if more than ten studies were pooled to avoid underpowered assessment [23].

### Data management and synthesis

Measurements were reported as means, the differences in means between samples, and variance (standard error, standard error of the mean or standard deviation). Studies reporting similar ultrasound measurements (similar technique and site) were pooled and statistical heterogeneity was calculated using *RevMan 5*.*3* [25]. If statistical heterogeneity was low (I^2^ <25%) [23, 26], random effects meta-analysis was used to calculate the difference in means (p<0.05) between participant groups of pooled studies. Sources of clinical and methodological heterogeneity and selection, performance, detection, attrition and reporting bias were identified by TM and KT independently, with disagreements resolved by consensus. Clinical heterogeneity was defined as variability in participant characteristics, co-existing conditions, co-interventions, outcomes evaluated and setting. Methodological heterogeneity was defined as variability in the study implementation, including ultrasound methodologies [23, 27].

## Results

### Search results

**Fig 1:**
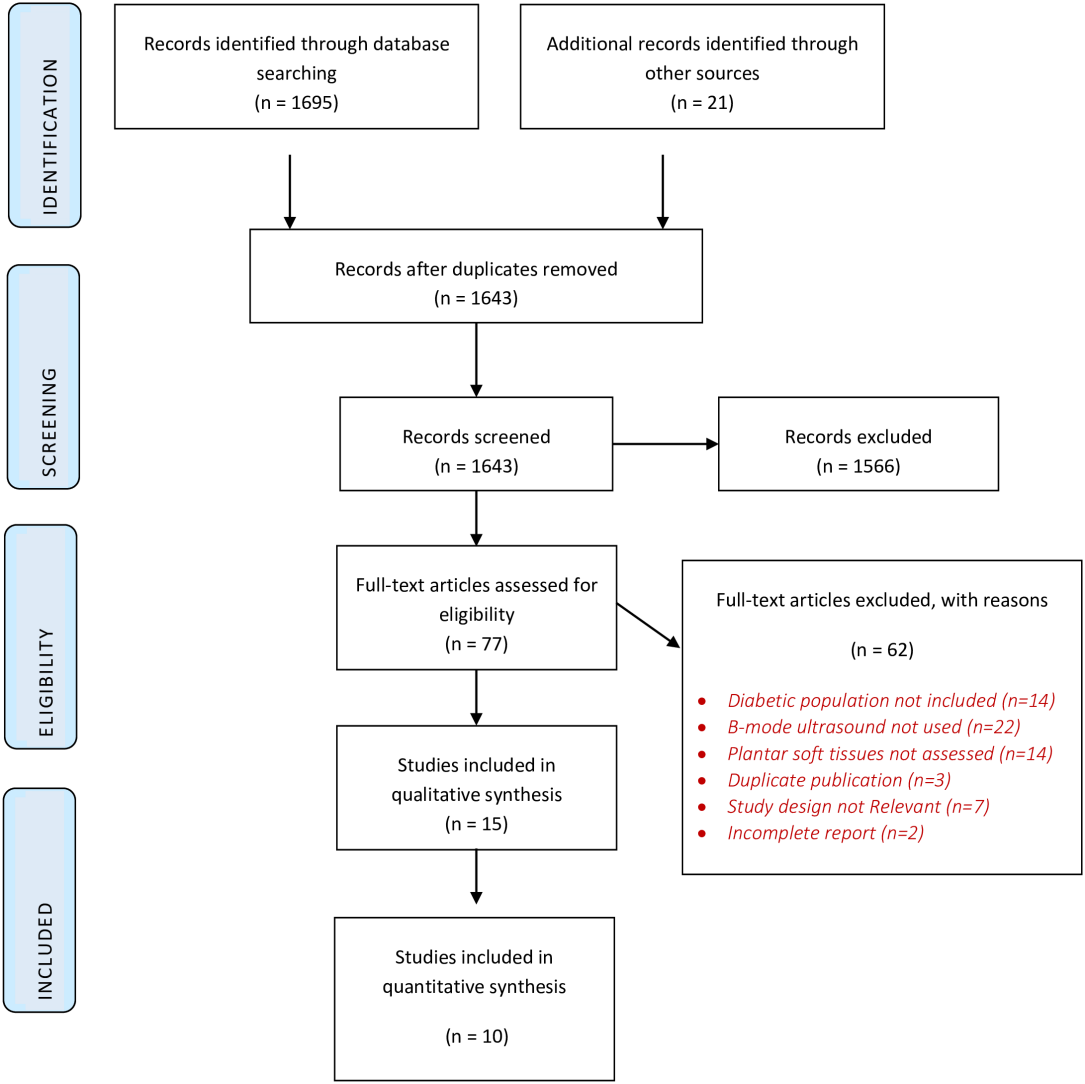
PRISMA flow diagram.

The database search revealed 1695 records (Fig 1). Twenty-one additional potential records were identified by manual searching. After duplicates were removed, articles were analysed for relevance with 77 records judged as potentially eligible. After full-text screening, 62 full-text articles were excluded resulting in the selection of 15 studies for data extraction and analysis. A third reviewer (SJ) was sought to adjudicate the eligibility for two studies.

### Study characteristics

Eligible studies were published between 1985 and 2015. Three studies were published after 2010. No studies used prognostic study designs.

Across the studies, 773 diabetic participants with varying disease chronicity and severity were included (Table 1). The most frequently reported duration of DM in groups containing participants with either mixed neuropathy or without peripheral neuropathy was six years (range 6 to 32 years). The average duration of DM for groups containing only diabetic peripheral neuropathy participants ranged from 15 to 32 years. We categorised DM participants with documented peripheral neuropathy or foot ulceration into Diabetic Group 2 and all other participants as Diabetic Group 1.

**Table 1:** Study & participant characteristics.

Study (Type)	Outcome Measures (ultrasound)	Tx Frequency (MHz)	Sample			DB Type	Duration Diabetes (years) *Mean (± SD)*		Mean Age, *years*, *Mean (± SD)*			Gender (M: F)			BMI (kg/m^2^)		
			NDB	DB Grp 1	DB Grp 2		DB Grp 1	DB Grp 2	NDB	DB Grp 1	DB Grp 2	NDB	DB Grp 1	DB Grp 2	NDB	DB Grp 1	DB Grp 2
**Kumar 2015 (CC)**	FPT sub-MTH, Skin-t, IFM	8–15	30	30 ^DB±^	DBPN+	T2	11.4 (5.48), DB±	11.4 (5.48), DB±	54 (9.86)	56 (6.63)		17:13	13:5	5:7	24.6 (1.7)	23.6 (2.74)	-
**Wang 2014 (CC)**	IFM	9–15	50	50 ^DBPN-^	56 ^DBPN+^	T2	6 (3), DBPN-	6 (3), DBPN-	59 (7)	59 (10)	63 (7) DBPN+	25:25	26:24	30:26	24.7 (5.8)	27.3 (7.1)	28.1 (4.7)
**Severinsen 2007 (CC)**	IFM	8–15	26	26 ^DB±^	DBPN+ only	T1/T2	32 (8–49), DB±	32 (8–49), DB±	49 (25–67)	49 (37–63)	47 (26–64) DBPN+	16:10	6:3	10:7	25.2c	23.5c	23.5c
**Chatzistergos 2014 (CC)**	TSD Heel Pad	13	17	35	-	T2	13.9 (7.8), DB*	13.9 (7.8), DB*	35 (5.8)	54.8 (9.1)	-	5:11^β^	27:8	-	25.9c	26.1c	-
**Hsu 2009 (CC)**	TSD Heel Pad	10	16	18	-	NS	6.88 (5.4), DB±	6.88 (5.4), DB±	55 (4.2)	57 (6.3)	-	9:7	7:11	-	24.3 (3.2)	26.7 (4.8)	-
**Petrofsky 2008 (CC)**	FPT, Skin-t MTH	10	15	10	-	NS	7.8 (3.5), DB*	7.8 (3.5), DB*	25.7 (2.89)	60.1 (5.7)	-	NS	NS	-	23.7c	35.3c	-
**Hsu 2007 (CC)**	TSD sub-MTH	5–12	8	13	-	T2	6.6 (SEM 2.5), DBPN-	6.6 (SEM 2.5), DBPN-	52.1(SEM 2.8)	53.4(SEM 2.6)	-	5:3	9:4	-	24.3 (SEM 1.1)	25.4 (SEM 1.3)	-
**Thomas 2003 (CC)**	TSD & Skin-Fascia 10 areas of plantar foot	7.5	18 feet (c 9 people)	36 feet ^DB±^ (c 18 people)	*5 feet (ulcer DBPN+)*	T2	10.6 (7.7) (DB±)	10.6 (7.7) (DB±)	45.4 (10.6)	56.77 (12.5)	-	6:3	12:6	-	-	-	-
**Tong 2003 (CC)**	TSD Heel Pad	5–12	14	9	-	T1/T2	9.7 (7.5), DB*	9.7 (7.5), DB*	43.2 (17)	58.8 (17)	-	6:8	5:4	-	22.6 (3.1)	26 (4.2)	-
**Duffin 2002 (CC)**	Skin-t	7	57	216	-	T1	6 (IQR3.9–10.2), DBPN-	6 (IQR3.9–10.2), DBPN-	15.6 (IQR 13.8–16.4)	15.3 (IQR 13.5–16.8)	-	27:30	101:115	-	NS	NS	-
**Abouaesha 2001 (CS)**	TSD sub-MTH	3.75		157 ^DBPN+^	-	T1/T2	16.4 (10.3), DBPN+	16.4 (10.3), DBPN+	-	61.2 (10.2)	-	-	73%:27% (115:42c)	-		29.3 (5.0)	-
**Hsu 2000 (CC)**	TSD Heel Pad	10	20	21^no ulcer^	12 ^ulcer^	T2	9.75 (8.0), no ulcer	9.75 (8.0), no ulcer	61 (50–74)	59 (40–77)	61 (50–74)	10/10	11:10	6:6	23.7 (3.0)	24.7 (3.0)	24.5 (6.1)
**Young 1995 (CC)**	TSD sub-MTH	NS	8	8 ^DB^	7 ^DBPN+^	NS	NS	NS	55 (30–64)	60 (41–68)	61 (44–68)	4:4	5:3	5:2	NS	NS	NS
**Gooding 1986 (CC)**	TSD Heel Pad & MTH	10	24	38 ^no ulcer^	11^ulcer^	NS	NS	NS	51 (3.8)	62 (1.2)	60 (2.3)	NS	NS	-	28.1c	27.1c	25.2c
**Gooding 1985 (CC)**	TSD Heel Pad	10	10	38		NS	NS	NS	28 (NS)	60 (30–77)		5:5	NS	-	NS	NS	-

Tx = Transducer, CC = Case-Control, CS = Cross-sectional, NDB = healthy control, DB = Diabetic, DBPN+ = Diabetic with peripheral neuropathy, DBPN- = Diabetic without peripheral neuropathy, DB± = Diabetic sample mixed DBPN- and DBPN+, IQR = Interquartile Range, SEM = Standard Error of Mean, SE = Standard Error, NS = not stated. c = calculated from data presented (i.e. not explicitly stated by authors). IFM = Intrinsic Foot Muscle, MTH = metatarsal head, TSD = Total soft tissue depth, FPT = Fat Pad Thickness, t = thickness, T1 = Type 1 Diabetes Mellitus, T2 = Type 2 Diabetes Mellitus, DB* = group neuropathy status not explicitly specified, IQR = Interquartile Range, SEM = Standard Error of Mean. β = one participant’s gender has not been provided.

All studies, excepting one [28], included a healthy non-diabetic group. Diabetes type was not explicitly stated in five studies. We categorised DM participant groups as: diabetic not defined (DB), diabetic with peripheral neuropathy (DBPN+), diabetic without peripheral neuropathy (DBPN-) and mixed diabetic group with and without neuropathy (DB±).

Ultrasound outcomes assessed were intrinsic foot muscle (IFM) dimensions [29–31], plantar heel pad thickness [32–38], forefoot soft tissue thickness [28, 30, 36, 38–40] and plantar skin thickness [30, 41, 42]. Distance and area measurements were standardised to millimetres (mm) or square millimetres (mm^2^) respectively. Where studies reported a standard error of the mean (SE or SEM), standard deviation (SD) was calculated using the formula $SD=SE\;x\;\sqrt{n}$ where *n* is the sample size. No studies reported outcome measurements assessing ultrasound morphologic soft tissue characteristics.

### Quality assessment

All studies used low level aetiologic study designs [18]; all were case-control design (level III-3) except one cross-sectional study [28] (level IV).

Studies were strong in stating study purpose, providing relevant background, describing the ultrasound procedure, using appropriate statistical analysis, reporting the clinical importance and making appropriate conclusions (S2 Table). Most studies described diabetic samples well, but control samples were poorly described, threatening internal validity. No study provided *a priori* power analysis. Overall, studies were moderately strong for reporting test-retest reliability, generalisability of the ultrasound procedure to a clinical setting (external validity) and using strategies to reduce confounding. Concurrent validity was weak; only two studies validated ultrasound measures against a reference standard [31, 37].

### Ultrasound measures

Results are presented for groups of similar ultrasound measurements. Across all groups, low bias was observed for only one category; reporting bias (S3 Table). Funnel plot analysis for publication bias was not performed due to the small number of studies in each grouping. An overview of ultrasound scan methods for plantar soft tissue outcome measures (skin and fat pad) is provided in S4 Table.

#### Skin thickness

Three studies measured plantar skin thickness [30, 41, 42]. Pooled samples included 102 non-diabetic and 256 DM participants (18 with neuropathy). Two studies [30, 41] reported thinner skin thickness in DM participants compared to one study [42] reporting no difference between DM and non-diabetic participants (Table 2). Statistical heterogeneity was very high (I^2^ 98%) across two studies reporting skin thickness at the same site (under first metatarsal head).

**Table 2:** Study results—Skin thickness.

Study	Measurement site	Interpretation of *skin* thickness *(e*.*g*. *Skin = Combined Epidermis & Dermis thickness)*	Skin thickness (mm) Mean ± SD		
			NDB (n)	DB (n)	p-value
**Kumar 2015**	Under all MTH’s	Not Defined	1MTH 2.4 ± 0.5 2MTH 2.6 ±0.4 3MTH 2.6 ± 0.4 4MTH 2.6 ± 0.4 5MTH 2.5 ± 0.46 (n = 30)	1MTH 1.7 ± 0.3 2MTH 1.9 ± 0.5 3MTH1.9 ± 0.5 4MTH 1.9 ± 0.6 5MTH 1.9 ± 0.7 (n = 30)	<0.001 (for all sites)
**Petrofsky 2008**	Ball of Foot	Not Defined	0.7 ± 0.2 (n = 15)	0.4 ± 0.1 (n = 10)	<0.05
**Duffin 2002**	Under 1^st^ MTH	Not Defined	1.0 ± 0.1 (n = 57)	1.0 ± 0.1 (n = 216)	↔

↔ = No Difference, NDB = healthy Control, DB = Diabetic, MTH = metatarsal head.

**Clinical heterogeneity**: Age, gender and BMI were evenly distributed across participant groups. Where stated, there was variability in the diabetic participants by neuropathic status and diabetic type (1&2).

**Methodologic heterogeneity**: Different measurement sites and inexplicit descriptions of landmarks to describe measurement planes were potential sources of heterogeneity. Duffin et al. [42] used a low-frequency transducer that may have compromised image resolution, measurement accuracy and precision. Petrofsky et al. [41] analysed a ‘ball of the foot’ measurement in combination with skin measurements at other body areas.

#### Heel pad thickness

Seven studies measured plantar heel pad thickness from skin to calcaneal cortex [32–38]. Pooled samples included 110 non-diabetic controls and 200 DM participants. Six studies reported measurements in the unloaded (non-weight bearing) state and one study [34] reported measurements in both unloaded and loaded (weight-bearing) states. Five studies reported non-statistically significant differences in the heel pad thickness between DM and non-diabetic participants [32–35, 38], one study reported thinner heel pad thickness in diabetics [36] and two reported thicker heel pad thickness in diabetics [34, 37] (Table 3). When comparing diabetic groups with and without peripheral neuropathy, one study reported no differences [35], and one [36] reported a thinner measurement in DM participants with ulceration.

**Table 3:** Study results–Heel pad thickness.

Study	Outcome Measure & Method	Result *mm*, *Mean ± SD*			Reported Outcome	Statistical significance (p-value)
		NDB (n)	DB Grp 1 (n)	DB Grp 2 (n)		
**Chatzistergos 2014**	TSD Unloaded	19.5 ± 4.7 (17)	19.4 ± 3.5 (35)	-	↔	0.985
**Hsu 2009**	TSD Unloaded	18.4 ± 12.0 (16)	19.3 ± 3.0 (18)	-	↔	0.222
**Thomas 2003**	TSD Unloaded	14.6 ± 3.1 (18 feet)	14.3 ± 1.5 (8 feet)	22.6 ± 0.0 (1 foot)	-	none reported (p>0.002)
**Tong 2003**	TSD Unloaded	15.5 ± 2.4 (14)	16.1 ± 2.4 (9)	-	↔	0.412
**Tong 2003**	TSD Loaded	10.1 ± 1.8 (14)	12.2 ± 2.3 (9)	-	↑t in DB	0.006
**Hsu 2000**	TSD Unloaded	16.5 ± 1.9 (20)	17.2 ± 3.1 (21^no ulcer)^	17.8 ± 3.7 (12 ^ulcer)^	↔	0.254
**Gooding 1986**	TSD Unloaded	18.62 (SE 0.36) (24)	17.33 (SE 0.29) (38 ^no ulcer)^	15.77 (SE 39^a^) (11 ^ulcer^)	↓t DB ^no ulcer^ vs NDB.	<0.01
					↓t DB ^ulcer^ vs DB ^no ulcer^ & NDB.	<0.01
**Gooding 1985**	TSD Unloaded	16.6 (SEM 0.32) (10)	17.8 (SEM 0.31) (38)	-	↑ t DB vs NDB	<0.01

TSD = Total soft tissue depth, Loaded = Weight bearing or compressed, Unloaded = Non-weight bearing / non-compressed, ↔ = No Difference, ↑ = Increased, ↓ = Decreased, t = Thickness, NDB = healthy Control, DB = Diabetic, SEM = Standard Error of Mean, SE = Standard Error,

^a^ Possible typographic error, most likely 0.39.

Statistical heterogeneity was high for studies reporting heel pad thickness (I^2^ 76–91%).

**Clinical heterogeneity**: Studies used strategies to limit potential confounders. BMI was evenly distributed across participant groups, but the age range was wider for non-diabetics, and gender distribution was not reported in all studies. There was variability in the neuropathic status and diabetes type of DM participants. Participant ethnicity, co-morbidities and co-interventions were not reported. Generalisability was weakened by inexplicit descriptions of control groups.

**Methodological heterogeneity**: There was wide variation in study conduct, measurement planes and landmarks and participant positioning. Two studies confirmed their measurements were reliable [33, 35] and one study [37] validated measurements against a reference standard.

#### Forefoot soft tissue thickness

Seven studies reported on sub-metatarsal head soft tissue thickness measurements [28, 30, 36, 38–41]. Thomas et al. [38] also reported on thickness at the hallux. Pooled samples included 94 healthy participants and 292 DM participants. One study [28] included only diabetic participants with peripheral neuropathy. Five studies measured the total soft tissue depth from skin to bone cortex (Table 4). Two studies [30, 41] measured fat pad thickness, excluding the joint capsule, tendons and skin. Petrofsky et al. [41] analysed their measurement at the ball of the foot in combination with measurements from other body areas.

**Table 4:** Study results–Forefoot tissue thickness.

Study	Outcome Measure, Site & State	Tissue Thickness *mm*, *Mean ± SD*			Reported Outcome	Statistical significance (p-value)
		NDB (n)	DB Grp 1 (n)	DB Grp 2 (n)		
**Kumar 2015**	FPT, 1–5 sub-MTH Unloaded	1MTH 4.9 ±1.2 2MTH 6.5 ±1.9 3MTH 6.3 ±1.6 4MTH 5.9 ±1.5 5MTH 5.4 ±1.3 (n = 30)	1MTH 3.9 ±1.1 2MTH 5.3 ±1.6 3MTH 5.2 ±1.4 4MTH 4.7 ±1.4 5MTH 4.2 ±1.1 (n = 30 ^DB±)^	-	↓t, p<0.05 (DB± vs NDB & DBPN- vs NDB @ all MTH), p<0.05 both,	<0.05
					↔ @ all MTH DBPN+ vs DBPN-p>0.05,	>0.05
					↔ DBPN+ vs NDB, p>0.05	
**Petrofsky 2008**	FPT, Ball of Foot Unloaded	1.4 ± 0.3 (n = 15)	0.8 ± 2.2 (n = 10)	-	↓ t DB vs NDB	<0.05
**Hsu 2007**	TSD, sub-MTH Unloaded	1MTH 14.1 (SEM 0.5) 2MTH 13.5 (SEM 0.7) 3MTH 12.6 (SEM 0.4) 4MTH 11.6 (SEM 0.3) 5MTH 11.2 (SEM 0.7) (n = 8)	1MTH 13.7 (SEM 0.6) 2MTH 13.4 (SEM 0.9) 3MTH 12.8 (SEM 0.9) 4MTH 12.7 (SEM 1.0) 5MTH 12.7 (SEM 0.6) (n = 13)	-	↔	>0.05
**Thomas 2003**	TSD** sub-MTH, Hallux Unloaded	2MTH 10.7 (1.4) (n = 18 feet)	2MTH 9.7 (1.3) (n = 9 feet)	2MTH 14.9 (-) (n = 1 foot)	-	Not reported (p>0.002)
		3-5MTH area 8.5 (1.8) (n = 18 feet)	3-5MTH area 8.3 (0.4) (n = 5 feet)	3-5MTH area 10.2 (-) (n = 1 foot)	-	Not reported (p>0.002)
		Hallux 6.4 (1.0) (n = 18 feet)	Hallux 5.9 (0.5) (n = 4 feet)	Hallux 10.4 (2.9) (n = 3 feet)	↑ t in DB ulcer (i.e. DBPN+) compared to NDB & DB no-ulcer	<0.005
**Gooding 1986**	TSD sub-MTH Unloaded	1MTH 12.92 (SE 0.42) 2MTH 14.17 (SE 0.26) 3MTH 13.56 (SE 0.29) 4MTH 12.91 (SE 0.34) 5MTH 11.47 (SE 0.27) (n = 24)	1MTH 11.6 (SE 0.29) 2MTH 12.7 (SE 0.29) 3MTH 13.06 (SE 0.24) 4MTH 12.1 (SE 0.26) 5MTH 10.7 (SE 0.23) (n = 38 ^no ulcer^)	1MTH 10.67 (SE 0.56) 2MTH 12.48 (SE 0.72) 3MTH 12.34 (SE 0.54) 4MTH 11.05 (SE 0.53) 5MTH 10.72 (SE 0.38) (n = 11^ulcer^)	↓t DB ^no ulcer^ vs NDB @ MTH1, MTH2.	<0.025
					↓t DB ^ulcer^ vs DB ^no ulcer^ & NDB @ MTH1, MTH2.	<0.05
**Abouaesha 2001**	TSD sub-MTH ^a^ Loaded	-	NO CALLUS 1MTH 11.1 ± 1.7 2MTH 9.1 ± 1.8 3MTH 8.1 ± 1.6 4MTH 7.6 ± 1.5 5MTH 5.9 ± 1.4 (157 ^DBPN+^)	CALLUS 1MTH 10.9 ± 1.4 2MTH 7.9 ± 1.7 3MTH 7.0 ± 1.4 4MTH 6.7 ± 1.3 5MTH 5.2 ± 1.2 (NS)	Callus vs No Callus = ↓ plantar tissue thickness in callus group. No sig diff btw L & R feet.	<0.001
**Young 1995**	TSD sub-MTH Loaded	1MTH 8.6 ± 1.7 2MTH 8.3 ± 2.3 3MTH 7.5 ± 1.8 4MTH 6.8 ± 1.5 5MTH 6.0 ± 2.4 (n = 8)	1MTH 10.7 ± 2.4 2MTH 8.6 ± 2.6 3MTH 7.6 ± 2.1 4MTH 6.9 ± 2.0 5MTH 6.7 ± 1.9 (n = 8 ^DB^)	1MTH 6.5 ± 2.6 2MTH 7.6 ± 2.1 3MTH 6.8 ± 1.5 4MTH 6.1 ± 1.6 5MTH 5.9 ± 1.4 (n = 7 ^DBPN+^)	NDB vs DBPN+ sig.↓t @ 1MTH only. No sig. difference at MTH 2–5	<0.005^b^
					DBPN+ vs DBPN- sig.↓t @ 1MTH only. No sig. difference at MTH 2–5	

TSD = Total soft tissue depth, FPT = Fat pad thickness, ↔ = No Difference, ↑ = Increased, ↓ = Decreased, t = Thickness, NDB = healthy Control, DB = Diabetic, DBPN+ = Diabetic with peripheral neuropathy, DBPN- = Diabetic without peripheral neuropathy, DB± = Diabetic sample mixed DBPN- and DBPN+, MTH = Metatarsal Head, SEM = Standard Error of Mean, SE = Standard Error, sig. = significance, Loaded = Weight bearing or compressed, Unloaded = Non-weight bearing / non-compressed.

^a^–Left foot values used. Results of both feet provided in report, there was negligible difference between tissue measurements for left and right feet with 0.3mm being the greatest difference between tissue measures at the 5^th^ metatarsal head. The left foot values were the greater dimensions and were used arbitrarily as a representation of the left and right values.

^b^–p-value provided for 1^st^ MTH, but this value is a combined analysis of variance across all groups (i.e., it is not specific to diabetic groups and includes continuous variable values from a rheumatoid metatarsalgia group).

**Sub-metatarsal head soft tissues**: Five studies [30, 36, 38, 39, 41] reported measurements in the unloaded (non-weight bearing) state and two [28, 40] reported measurements in loaded (weight-bearing) states. All six studies comparing measurements between healthy and DM groups [30, 36, 38–41] reported significantly thinner thickness in DM participants (p< 0.05), except two [38, 39] (Table 4). Thinning was reported at different sites; under the first metatarsal head [30, 36, 40], the second metatarsal head [30, 36] and under metatarsal heads 3–5 [30]. Soft tissues were thinner under all metatarsal heads in participants with more chronic disease [36, 40] but only reaching statistical significance under the first [36, 40] and second [36] metatarsal heads. Abouaesha et al. [28] compared participants with and without callus, reporting thinner loaded plantar tissue thickness in participants with callus (p<0.001).

**Hallux**: Thomas et al. [38] reported a significant increase in the soft tissue thickness at the hallux (p<0.005) in a diabetic sample with ulceration compared to a control group and DM group without ulceration.

**Clinical heterogeneity**: Age and BMI were evenly distributed and there was a greater male prevalence across participant groups. There was variability in the diabetic participants by neuropathic status and diabetic type.

**Methodological heterogeneity**: There was variability in study design, participant positioning and measurement sites, planes and landmarks. Abouaesha et al. [28] used a transducer frequency with poor resolution, raising measurement precision concerns. Across studies, measurements had not been validated against a reference standard and the ultrasound procedure was generalisable to standard clinical settings in only two studies [30, 36].

**Fig 2:**
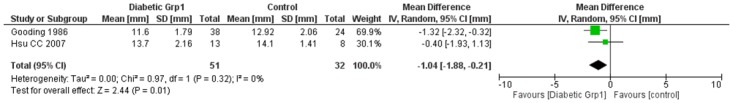
Meta-analysis and forest plot for sub-metatarsal head 1 unloaded total soft tissue depth, non-diabetic and diabetic group 1.

**Fig 3:**
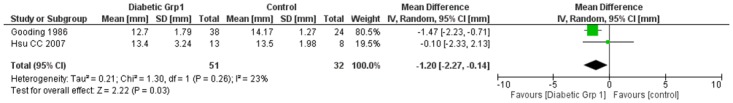
Meta-analysis and forest plot for sub-metatarsal head 2 unloaded total soft tissue depth, non-diabetic and diabetic group 1.

**Fig 4:**
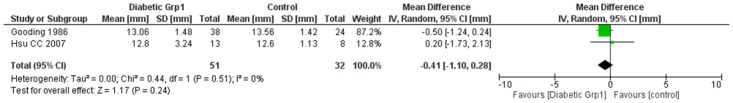
Meta-analysis and forest plot for sub-metatarsal head 3 unloaded total soft tissue depth, non-diabetic and diabetic group 1.

**Fig 5:**
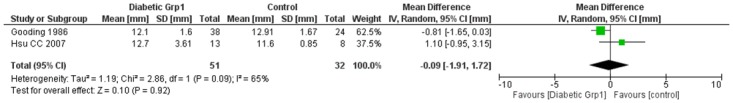
Meta-analysis and forest plot for sub-metatarsal head 4 unloaded total soft tissue depth, non-diabetic and diabetic group 1.

**Fig 6:**
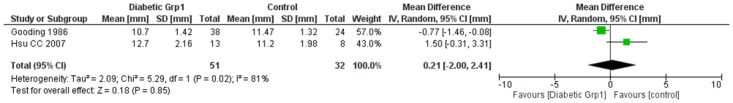
Meta-analysis and forest plot for sub-metatarsal head 5 unloaded total soft tissue depth, non-diabetic and diabetic group 1.

Statistical heterogeneity was high across studies measuring soft tissue thickness under metatarsal heads four and five (I^2^ 65%, 81%). Statistical heterogeneity was low for metatarsal heads one—three (I^2^ 0%, 23%, 0% respectively). A meta-analysis of two studies that measured the unloaded total soft tissue depth between non-diabetic and diabetic (no ulcer) groups [36, 39] at metatarsal heads one—three (Figs 2–6) demonstrated a reduction in tissue thickness in DM participants compared to non-diabetics under the first metatarsal head (95% CI -1.88, -0.21mm, p = 0.01), the second metatarsal head (95% CI -2.27, -0.14mm, p = 0.03), but not under the third metatarsal head.

#### Intrinsic foot muscles (IFM)

Three studies reported IFM dimensions [29–31]. Pooled samples included 106 non-diabetic control and 162 DM participants. All studies reported thickness and cross-sectional dimensions of the extensor digitorum brevis muscle (EDB), two reported a combined muscle measurement in the first metatarsal interspace [29, 31] and one study [30] reported discrete first interspace muscle dimensions (first dorsal interosseous, first lumbrical and the adductor hallucis muscle).

EDB was measured by thickness, transverse diameter and CSA. EDB dimensions were consistently significantly thinner in diabetics for all dimension measurements of EDB compared to healthy controls except for one study [30], which reported no significant differences in EDB thickness (Table 5).

**Table 5:** Study results–Intrinsic foot muscles.

Study	Ultrasound Measurement	Group *n*				*Dimensions*, *Mean ± SD*					
		NDB	DB Grp 1	DB Grp 2	DB Grp 3	NDB	DB Grp 1	DB Grp 2	DB Grp 3	Statistical significance (p-value)	
**Kumar 2015**	EDB t (mm) csa (mm^*2)*^	30	30 ^DB±^	DBPN+ only	-	-7.7 ± 1.0 -217 ± 42	-7.3 ± 2.1 -172 ± 42	-	-	DB± vs NDB	t, 0.31 csa, <0.001
										DBPN- vs NDB	t, p = 0.070 csa, 0.002
										DBPN+ vs NDB	csa, 0.012
										DBPN+ vs DBPN-	t, 0.926 csa, 0.985
**Wang 2014**		50	50 ^DBPN-^	56 ^DBPN+^	-	7.16 ± 0.94 165.42 ± 32.86	6.91 ± 0.97 138.1± 39.26	5.61 ± 0.90 90.4± 29.9	-	DBPN- vs NDB	csa, <0.01
										DBPN+ vs NDB	t, csa, <0.01
										DBPN+ vs DBPN-	t, csa, <0.01
**Severinsen 2007**		26	26 ^DB±^	DBPN+ only	-	-9.0 ± 1.0 -214 ± 38	-6.4 ± 2.1 -116 ± 65	-5.8 ± 2.1	-	DB± vs NDB	t, csa, <0.001
										DBPN+ vs NDB	-
										DBPN+ vs DBPN-	t, <0.05
**Wang 2014**	EDB Transverse diameter (mm)	50	50 ^DBPN-^	56 ^DBPN+^	-	75.84 ± 9.03	66.93 ± 9.28	53.95 ± 11.05	-	DBPN- vs NDB	<0.01
										DBPN+ vs NDB	<0.01
										DBPN+ vs DBPN-	<0.01
**Kumar 2015**	1^st^ Lumbrical t (mm)	30	30 ^DB±^	DBPN+ only	-	15.7 ± 5.0	12.9 ± 4.4	-	-	DB± vs NDB	0.02
										DBPN- vs NDB	0.006
										DBPN+ vs NDB	0.961
										DBPN+ vs DBPN-	0.059
**Kumar 2015**	1^st^ Interosseous t (mm)				-	15.7 ± 5.0	12.9 ± 4.5	-	-	DB± vs NDB	0.02
										DBPN- vs NDB	0.006
										DBPN+ vs NDB	0.964
										DBPN+ vs DBPN-	0.058
**Kumar 2015**	Adductor Hallucis t (mm)				-	15.3 ± 4.3	10.5 ± 1.3	-	-	DB± vs NDB	0.001
										DBPN- vs NDB	-0.001
										DBPN+ vs NDB	0.002
										DBPN+ vs DBPN-	0.965
**Wang 2014**	MIL t (mm)	50	50 ^DBPN-^	56 ^DBPN+^	-	34.32 ± 1.93	32.16 ± 2.86	30.7 ± 2.85	-	DBPN- vs NDB	<0.01
										DBPN+ vs NDB	<0.01
										DBPN+ vs DBPN-	<0.01
**Severinsen 2007**		26	26 ^DB±^	DBPN+ only	DBPN- only	40.2 ± 3.2	29.6 ± 8.3	28.3 ± 8.8	35.6 ± 4.3	DB± vs NDB	<0.001
										DBPN+ vs DBPN-	<0.03

t = thickness (mm), csa = cross-sectional area (mm^2^), NDB = healthy Control, DB = Diabetic, DBPN+ = Diabetic with peripheral neuropathy, DBPN- = Diabetic without peripheral neuropathy, DB± = Combined diabetic sample mixed DBPN- and DBPN+, EDB = Extensor digitorum brevis muscle, MIL = *combined thickness 1st Dorsal Interosseous + Adductor hallucis + 1st Lumbrical muscles*.

Two studies reporting the combined thickness of the 1st dorsal interosseous, adductor hallucis and 1st lumbrical muscles reported significantly thinner values in diabetics compared to healthy controls, and thinner values in DM participants with peripheral neuropathy (Table 5).

Statistical heterogeneity was high across three studies reporting CSA and thickness of the EDB (I^2^ 91%, 89%).

**Clinical heterogeneity**: Age and BMI ranges were similar between healthy and diabetic groups. Two studies matched for gender. Across studies, participants had different DM type and neuropathic status.

**Methodologic heterogeneity**: Differences in ultrasound measurement methods and small sample sizes were potential sources of heterogeneity. One study [31] validated their measurement against a reference standard (MRI).

## Discussion

There is merit in investigating the value of ultrasound measurements of the plantar soft tissues as a predictor of diabetes-related change as its low cost, low risk, portability, non-invasiveness and accessibility would suit community screening programs. We identified fifteen studies that compared dimensional ultrasound measurements of the soft tissue of the foot between diabetic and non-diabetic participants, and between different groups of diabetic participants including those with and without peripheral neuropathy or foot ulceration. A range of plantar foot structures were investigated including the skin, heel pad, forefoot and intrinsic muscles. While some studies demonstrated that ultrasound identified structural changes in the soft tissues of the diabetic foot compared to a non-diabetic foot, the study designs are not strong enough to suggest these changes are either causal or predictive of diabetes-related foot complications.

Comparisons of ultrasound measures of plantar skin and heel pad thickness between people with and without DM and between people with different DM duration were inconsistent across studies. Participants with more chronic DM were under represented in studies measuring skin thickness; only 18 of the 256 diabetic participants had diabetic peripheral neuropathy which limited generalisability across the spectrum of the diabetic population.

For studies reporting measurements of intrinsic foot muscles, and despite the wide variation in the methods used to measure them, dimensions were smaller in participants with diabetes compared to healthy controls, except for one study [30]. This study reported no differences in measurements of EDB thickness between controls and people with DM (mixed DM sample with and without neuropathy), and in measurements of the thickness of the 1^st^ interosseous and 1^st^ lumbrical muscles between controls and people with neuropathic DM. Similarly, when intrinsic muscle dimensions were compared between diabetic groups, smaller dimensions were noted in the more chronic groups, except one study [30] that was potentially underpowered for such an analysis. These findings are consistent with a MRI study [8] which reported that neuropathic diabetic people had nearly half the total intrinsic muscle volume of healthy people and people without diabetic neuropathy. The CSA of the EDB most consistently demonstrated differences between those with and without diabetes, and between diabetic people of differing neuropathy status, suggesting it may be a predictor of diabetes-related changes in the foot. However, there are technical challenges in assessing the intrinsic muscles using ultrasound. Differentiation of each muscle can be difficult, particularly in muscles with fatty replacement or atrophy. This is increasingly likely with progressing diabetic chronicity and age or immobilisation [43].

Measurements of the soft tissues under the first and second metatarsal heads are also potential predictors of future diabetic soft tissue changes, with the consistent finding of significantly thinner soft tissues in diabetic compared to non-diabetic participants and diabetic participants with peripheral neuropathy compared to those without peripheral neuropathy, and confirmed in a meta-analysis across two studies [36, 39]. These measurements were demonstrated to be reliable [39], although this was achieved using a unique apparatus not readily available in clinical settings. Careful consideration of the precise site of this measurement at the first metatarsal head is required before adoption. The measurement will differ depending whether it is performed overlying either of the two sesamoid bones or directly over the first metatarsal head and between the sesamoids. Averaging measurements made at each of the medial and lateral sesamoids is another approach which has been reported to improve correlation between plantar tissue thickness and peak plantar pressure compared with measurement of tissue thickness directly over the first metatarsal head [28]. In addition to investigating the best measurement technique, future investigations of measurements of the soft tissue dimensions at the first metatarsal should include testing of reliability, validity and predictive value.

We identified three studies that included a diabetic group with foot ulceration [35, 36, 38]. They reported statistically significant differences when comparing ulcer and non-ulcer comparison groups, although there was little consistency regarding the actual dimension changes. This discordance was due to methodological differences, small chronic DM sample sizes and differences in the approach each study used for measuring soft tissue thicknesses in feet with ulcers.

High statistical heterogeneity across studies using similar measurement techniques at the same anatomic site was probably due to sampling and study design biases, and variations in ultrasound methodology and clinical populations. Confounder reducing strategies were also limited across studies. In separate studies, Prichasuk et al. [44] and Campanelli et al. [45] demonstrated that heel pad thickness is greater in men than women, suggesting gender is a confounder. Across-studies, there were often gender imbalances with less than a third of studies matching for gender increasing the risk of within-study gender bias.

Ultrasound measurement techniques, including landmarks used to make measurements and the transducer plane, potentially impacted on methodological heterogeneity. Ultrasound imaging is highly operator dependent requiring an extensive knowledge of anatomy, imaging artefacts, anatomical variants and ultrasound technique [46]. Unskilled operators will therefore increase the potential for measurement and interpretation errors. No study included explicit reporting of experience and training of the ultrasound operator. Additionally, two studies used inappropriate transducers for optimal resolution. Abouaesha et al. [28] used a curvilinear 3.5MHz transducer, which is not suited to musculoskeletal ultrasound as it lacks near-field resolution and can have contact difficulties due to the curved aperture. Duffin et al. [42] used a transducer frequency that was too low (7MHz) for measuring skin thickness.

This review is limited by heterogeneity across studies, the absence of cohort or randomised control studies and weak quality. Where meta-analysis could be undertaken across homogenous studies, it was limited to two studies, restricting the review to mostly narrative analysis. Sample populations of participants with peripheral neuropathy were underrepresented and heterogenous between studies limiting inferences across the full spectrum of the diabetic population.

## Conclusion

This review has not revealed direct evidence to support the use of high resolution (B-mode) ultrasound to identify soft tissue changes of the foot as a cause or predictor of diabetes-related complications in the foot. However, the finding that dimensions of soft tissues under the first and second metatarsal heads and the EDB muscles are reduced in diabetic people compared to healthy people, and is further reduced in people with more chronic diabetes, suggests that standardised and reproducible ultrasound measurement techniques may have a role in identifying causal or predictive changes and which could be tested with quality prospective cohort studies.

## Supporting information

S1 Fig PICOS search strategy. (PDF)

S2 Fig Elements of risk of bias defined (adapted Cochrane Handbook & Viswanathan and colleagues (2012)). (PDF)

S3 Fig Assessment of risk of bias defined (adapted Higgins and Green (2011) & Viswanathan and colleagues (2012)). (PDF)

S4 Fig PRISMA checklist. (DOC)

S1 Table Example search strategy. (PDF)

S2 Table McMaster critical review summary (as determined by reviewers). (DOCX)

S3 Table Assessment of risk of bias outcomes: Summary of magnitude of overall bias (as determined by reviewers). (DOCX)

S4 Table Ultrasound scanning methods for plantar soft tissue outcome measures. (DOCX)
