# Supplementary material for: Can ultrasound measures of intrinsic foot muscles and plantar soft tissues predict future diabetes-related foot disease? A systematic review
Source: PLoS One. 2018 Jun 15;13(6):e0199055. doi: 10.1371/journal.pone.0199055 (PMC6003689; doi:10.1371/journal.pone.0199055)
Supplement: S1 Fig — (PDF) [file pone.0199055.s001.pdf]

S1 Fig

**P**	Participants	Humans with Type 1 diabetes mellitus, Type 2 diabetes mellitus.
**I**	Intervention (Exposures)	Diabetes (no restrictions on chronicity, type or duration).
**C**	Control (comparison)	Humans who do not have diabetes.
**O**	Outcomes	Outcomes were B-mode ultrasound measures of the dimensions or morphology of plantar soft tissue structures and intrinsic muscles of the foot.
**S**	Studies Included	All aetiological and prognostic study designs (according to NHMRC*) that were performed in accordance with the Declaration of Helsinki ethical standards.

*National Health and Medical Research Council (NHMRC) [1-3]

1. NHMRC. How to review the evidence : systematic identification and review of the scientific literature : handbook series on preparing clinical practice guidelines. Canberra: National Health and Medical Research Council; 2000.
2. Coleman K, Norris S, Weston A, Grimmer-Somers K, Hillier S, Merlin T, et al. NHMRC additional levels of evidence and grades for recommendations for developers of guidelines: Stage 2 consultation: National Health and Medical Research Council (NHMRC); 2008.
3. Merlin T, Weston A, Tooher R. Extending an evidence hierarchy to include topics other than treatment: revising the Australian 'levels of evidence'. BMC Medical Research Methodology. 2009;9(1):34. doi: 10.1186/1471-2288-9-34.
