# Supplementary material for: Can ultrasound measures of intrinsic foot muscles and plantar soft tissues predict future diabetes-related foot disease? A systematic review
Source: PLoS One. 2018 Jun 15;13(6):e0199055. doi: 10.1371/journal.pone.0199055 (PMC6003689; doi:10.1371/journal.pone.0199055)
Supplement: S2 Fig — (PDF) [file pone.0199055.s002.pdf]

S2 Fig

Fundamental Bias Elements	Assessment Criteria	Assessment Criteria Defined
**Selection**	Baseline characteristics of the sample groups are comparable.	Sample selection and recruitment processes are described and exposure (disease) and control groups have similar baseline characteristics (i.e. the sample is generalizable). Baseline characteristics include demographic, clinical and social characteristics.
	Sample bias.	Volunteer or referral bias, healthy worker bias, Berkson's bias, non-response bias.
**Performance**	Level of care provided equally between groups.	Co-interventions such as other podiatric care and unintended treatments taken into consideration and described.
**Attrition**	Completeness of outcome data.	Systematic differences in the loss of participants from the study and were they appropriately accounted for in the statistical analysis.
**Detection**	Consistency and blinding of the outcome assessors.	Were steps in place to avoid differences in outcomes assessment and potential for assessor to measure in favour of the expected outcome (i.e. blinding).
	Bias in internal statistics.	Faulty or erroneous statistical analysis that affects validity of effect estimates.
	Valid and reliable measures.	Were outcome measurement techniques deemed accurate and was ultrasound reliability tested.
**Reporting**	Selective outcome reporting.	Systematic differences between outcomes described in methodology discussion and the results reported.

1. Higgins J, Green S. Cochrane Handbook for Systematic Reviews of Interventions Version 5.1.0. The Cochrane Collaboration; 2011.

2. Viswanathan M, Ansari M, Berkman N, Chang S, Hartling L, McPheeters L, et al. Assessing the Risk of Bias of Individual Studies in Systematic Reviews of Health Care Interventions. 2012. In: Methods Guide for Effectiveness and Comparative Effectiveness Reviews [Internet]. Rockville (MD): Agency for Healthcare Research and Quality. Available from: www.effectivehealthcare.ahrq.gov/.
