# Supplementary material for: Can ultrasound measures of intrinsic foot muscles and plantar soft tissues predict future diabetes-related foot disease? A systematic review
Source: PLoS One. 2018 Jun 15;13(6):e0199055. doi: 10.1371/journal.pone.0199055 (PMC6003689; doi:10.1371/journal.pone.0199055)
Supplement: S3 Fig — (PDF) [file pone.0199055.s003.pdf]

S3 Fig:

	Sampling Bias		Performance Bias	Attrition	Detection Bias			Reporting Bias
	Consecutive or randomised sampling described (Y / N)	Baseline characteristics of the groups are comparable	Level of podiatric care provided equally between groups	Completeness of outcome data	Blinding of outcome assessors<br>(ULTRASOUND MEASURES)	Bias in Internal Statistics	Valid & Reliable Outcome Measures	Selective Outcome reporting
**L**<br>***Low overall risk of bias that is unlikely to significantly impact the results***	Sequencing (sequence generation) of sample specified.	The study describes the sample selection and recruitment process in detail and the groups (exposure and control) have *similar* characteristics and demographics. I.e. are generalizable.	All sample groups received the same level of podiatric care, attention and interventions (non-study related).	Loss of participants from the study were discussed and appropriately accounted for in the statistical analysis.<br>OR<br>It is perceived that there is no reasonable affect from attrition.	The operator performing the ultrasound measurements was blinded to the disease state and real-time measurements.	Appropriate statistical methods and analysis.	Ultrasound measurement techniques and the equipment used were appropriate.	All outcome measures were reported and addressed in appropriate detail.
**U**<br>***Unclear risk of bias with potential to alter results***	Limited discussion	There is insufficient information to make an informed judgement. Sample selection, recruitment, group characteristics and other comparable information is absent or limited.	There is limited or insufficient information to make an informed judgement.	There is limited or insufficient information to make an informed judgement about attrition.	There is limited or insufficient information to make an informed judgement.	There is limited or insufficient information to make an informed judgement.<br>OR<br>Both feet have been used for data collection per participant. It is unclear if allowances for this have been made in statistical analysis (i.e. potential for multiplicity bias.	Ultrasound measurements techniques were not described in sufficient detail or the transducer used was not of an appropriate frequency.<br>OR<br>Specific methods of ultrasound outcome measures were vague.<br>OR<br>Ultrasound reliability was not tested.	All outcome measures have been addressed to some extent but difficult to determine if addressed in sufficient detail.<br>OR<br>Incomplete outcome measure assessments; adequate and logical explanation has been provided as to why.
**S**<br>***significant risk of bias resulting in reduced confidence in results***	No discussion regarding sequence generation	It is clear that the sample / groups are not comparable and not generalizable.	Sample groups received differing levels of non-study related podiatric care.	Significant loss of participants without acknowledgement or accountability in statistical analysis.<br>OR<br>Significant attrition effect despite statistical allowances.	The operator performing the ultrasound measurements was clearly NOT blinded to the disease state and real-time measurements.	Faulty or erroneous statistical analysis that affects validity of effect estimates.	Errors in ultrasound measurements of described variables that would affect validity and estimates.	All outcome measures have NOT been addressed and no explanation has been provided as to why.

1. Higgins J, Green S. Cochrane Handbook for Systematic Reviews of Interventions Version 5.1.0. The Cochrane Collaboration; 2011.

2. Viswanathan M, Ansari M, Berkman N, Chang S, Hartling L, McPheeters L, et al. Assessing the Risk of Bias of Individual Studies in Systematic Reviews of Health Care Interventions. 2012. In: Methods Guide for Effectiveness and Comparative Effectiveness Reviews [Internet]. Rockville (MD): Agency for Healthcare Research and Quality. Available from: www.effectivehealthcare.ahrq.gov/.
