# Supplementary material for: Can ultrasound measures of intrinsic foot muscles and plantar soft tissues predict future diabetes-related foot disease? A systematic review
Source: PLoS One. 2018 Jun 15;13(6):e0199055. doi: 10.1371/journal.pone.0199055 (PMC6003689; doi:10.1371/journal.pone.0199055)
Supplement: S1 Table — (PDF) [file pone.0199055.s005.pdf]

S1 Table

Database	Search String
PubMed	(("diabetes mellitus"[mesh] OR "diabetes mellitus"[mh] OR ("diabetes mellitus"[MeSH Terms] OR ("diabetes"[All Fields] AND "mellitus"[All Fields]) OR "diabetes mellitus"[All Fields] OR "diabetes"[All Fields] OR "diabetes insipidus"[MeSH Terms] OR ("diabetes"[All Fields] AND "insipidus"[All Fields]) OR "diabetes insipidus"[All Fields]) OR diabetic[All Fields]) AND ("foot"[MeSH Terms] OR "foot"[MeSH Terms] OR ("foot"[MeSH Terms] OR "foot"[All Fields] OR "feet"[All Fields]) OR ("heel"[MeSH Terms] OR "heel"[All Fields]) OR "heel pad"[All Fields] OR heel-pad[All Fields] OR "fat pad"[All Fields] OR "plantar fat pad"[All Fields] OR "plantar fat-pad"[All Fields] OR (sub-metatarsal[All Fields] AND ("adipose tissue"[MeSH Terms] OR ("adipose"[All Fields] AND "tissue"[All Fields]) OR "adipose tissue"[All Fields] OR ("fat"[All Fields] AND "pad"[All Fields]) OR "fat pad"[All Fields])) OR (sub-metatarsal[All Fields] AND ("adipose tissue"[MeSH Terms] OR ("adipose"[All Fields] AND "tissue"[All Fields]) OR "adipose tissue"[All Fields] OR ("fat"[All Fields] AND "pad"[All Fields]) OR "fat pad"[All Fields])) OR "sub-metatarsal pad"[All Fields] OR "sole of the foot"[All Fields] OR "plantar soft tissues"[All Fields] OR "intrinsic muscles"[All Fields] OR "foot muscle"[All Fields] OR "foot muscles"[All Fields])) AND ("ultrasonography"[MeSH Terms] OR "ultrasonography"[MeSH Terms] OR ("ultrasonography"[Subheading] OR "ultrasonography"[All Fields] OR "ultrasound"[All Fields] OR "ultrasonography"[MeSH Terms] OR "ultrasound"[All Fields] OR "ultrasonics"[MeSH Terms] OR "ultrasonics"[All Fields]) OR ("ultrasonography"[MeSH Terms] OR "ultrasonography"[All Fields] OR "sonography"[All Fields]) OR "diagnostic ultrasound"[All Fields] OR "diagnostic sonography"[All Fields] OR "medical sonography"[All Fields] OR "ultrasound imaging"[All Fields] OR ("ultrasonography"[Subheading] OR "ultrasonography"[All Fields] OR "echography"[All Fields] OR "ultrasonography"[MeSH Terms] OR "echography"[All Fields]))
Medline	(diabetes mellitus/ or diabetes mellitus, type 1/ or diabetes mellitus, type 2/) OR (diabet*.mp. [mp=title, abstract, original title, name of substance word, subject heading word, keyword heading word, protocol supplementary concept word, rare disease supplementary concept word, unique identifier])) AND ((((heel or plantar or fat pad or fatpad or sole or muscle or panniculus carnosus or subcutaneous or submetatarsal or sub-metatarsal or microchamber or micro chamber or macrochamber or macro chamber) and (foot or feet)).mp. [mp=title, abstract, original title, name of substance word, subject heading word, keyword heading word, protocol supplementary concept word, rare disease supplementary concept word, unique identifier]) OR (exp foot)) AND ((Ultrasonography/) OR ((ultrasonography or ultrasound or sonography or medical sonography or diagnostic sonography or medical ultrasound or diagnostic ultrasound).mp. [mp=title, abstract, original title, name of substance word, subject heading word, keyword heading word, protocol supplementary concept word, rare disease supplementary concept word, unique identifier]))
