# Supplementary material for: Can ultrasound measures of intrinsic foot muscles and plantar soft tissues predict future diabetes-related foot disease? A systematic review
Source: PLoS One. 2018 Jun 15;13(6):e0199055. doi: 10.1371/journal.pone.0199055 (PMC6003689; doi:10.1371/journal.pone.0199055)
Supplement: S2 Table — (DOCX) [file pone.0199055.s006.docx]

**S2 Table**

| Citation | **Study Purpose** | **Literature** | **Design** | | | **Sample** | | | **Outcomes** | | | | | **Intervention** | **Results** | | | **Conclusions and Implications** |
| --- | --- | --- | --- | --- | --- | --- | --- | --- | --- | --- | --- | --- | --- | --- | --- | --- | --- | --- |
|  | Clearly stated | Relevant background literature | Study type | Appropriate | Level of evidence | Described in detail | Size justified | Strategies to reduce confounding | Outcome measures reliable | Outcome measures valid | Concurrent validity | External validity | Internal validity | Described in detail | Reported in terms of significance | Analysis method appropriate | Clinical importance reported | Conclusions appropriate |
| Kumar 2015 | Y | Y | CC | Y | III-3 | Y | N | Lim | NS | NS | N | Y | NS | Y | Y | Y | Y | Y |
| Wang 2014 | Y | Y | CC | Y | III-3 | Y | N | Y | NS | Y | N | Y | N | Y | Y | Y | Y | Y |
| Severinsen 2007 | Y | Y | CC | Y | III-3 | Y | N | Y | Y | Y | Y | Y | Y | Y | Y | Y | Y | Y |
| Chatzistergos 2014 | Y | Y | CC | Y | III-3 | Y | N | N | NS | Y | N | N | N | Y | Y | Y | Y | Y |
| Hsu 2009 | Y | Y | CC | Y | III-3 | Y | N | Y | Y | Y | N | Y | N | Y | Y | Y | Y | N |
| Petrofsky 2008 | Y | Y | CC | Y | III-3 | N | N | N | NS | NS | N | N | N | N | Y | Y | Y | Y |
| Hsu 2007 | Y | Y | CC | Y | III-3 | Y | N | Y | Y | Y | N | N | N | Y | Y | Y | Y | Y |
| Thomas 2003 | Y | Y | CC | Y | III-3 | N | N | N | N | N | N | N | N | N | Y | Y | Y | Y |
| Tong 2003 | Y | Y | CC | Y | III-3 | Y | N | N | NS | Y | N | N | N | Y | Y | Y | Y | Y |
| Duffin 2002 | Y | Y | CC | Y | III-3 | Y | N | Y | Y | Y | N | Y | Y | Y | Y | Y | Y | Y |
| Abouaesha 2001 | Y | Y | CS | Y | IV | Y | N | - | Y | Y | N | N | N | Y | Y | Y | Y | Y |
| Hsu 2000 | Y | Y | CC | Y | III-3 | Y | N | Y | Y | Y | N | Y | N | Y | Y | Y | Y | Y |
| Young 1995 | Y | Y | CC | Y | III-3 | N | N | Lim | Y | Y | N | N | N | N | Y | Y | Y | Y |
| Gooding 1986 | Y | Y | CC | N | III-3 | Y | N | Lim | NS | Y | N | Y | N | Y | Y | NS | Y | Y |
| Gooding 1985 | Y | Y | CC | Y | III-3 | N | N | N | NS | Y | Y | Y | N | N | Y | NS | Y | Y |

Y= Yes, N= No, NS = Not Specified (Not Sufficiently Addressed), Lim=Limited, CC=case-control, CS=cross-sectional.
