# Supplementary material for: Can ultrasound measures of intrinsic foot muscles and plantar soft tissues predict future diabetes-related foot disease? A systematic review
Source: PLoS One. 2018 Jun 15;13(6):e0199055. doi: 10.1371/journal.pone.0199055 (PMC6003689; doi:10.1371/journal.pone.0199055)
Supplement: S3 Table — (DOCX) [file pone.0199055.s007.docx]

**S3 Table**

| Study | Outcome Measure | Sampling Bias | | Performance Bias | Attrition | Detection Bias | | | | Reporting Bias |
| --- | --- | --- | --- | --- | --- | --- | --- | --- | --- | --- |
|  |  | Consecutive or randomised sampling described  (Y / N) | Baseline characteristics of the groups are comparable | Level of podiatric care provided equally between groups | Completeness of outcome data | Blinding of Outcome Assessors | Bias in Internal Statistics | Outcome Measures Reliable | Outcome Measures Valid | Selective Outcome reporting |
| PLANTAR SKIN | | | | | | | | | | |
| **Kumar 2015** | THICKNESS | N | U | U | L | U | L | U | U | L |
| **Petrofsky 2008** | THICKNESS | N | S | U | L | U | L | U | U | L |
| **Duffin 2002** | THICKNESS | N | L | L | L | L | L | L | L | L |
| HEEL PAD THICKNESS | | | | | | | | | | |
| **Chatzistergos 2014** | TSD | N | U | U | L | U | L | U | L | L |
| **Hsu 2009** | TSD | N | U | U | U | U | U | L | U | L |
| **Thomas 2003** | Skin-bone, Skin-fascia UL | N | U | U | U | U | L | U | U | L |
| **Tong 2003** | TSD (UL + L) | N | U | U | L | U | U | U | L | L |
| **Hsu 2000** | TSD | N | U | U | L | U | U | L | L | L |
| **Gooding 1986** | TSD | N | U | U | U | U | U | U | L | L |
| **Gooding 1985** | TSD | N | U | U | L | U | U | U | L | L |
| FOREFOOT | | | | | | | | | | |
| **Kumar 2015** | FPT, MTH | N | U | U | L | U | L | U | U | L |
| **Petrofsky 2008** | FPT, MTH | N | S | U | L | U | L | U | U | L |
| **Hsu 2007** | TSD, MTH | N | U | U | L | U | L | L | L | L |
| **Thomas 2003** | Skin-bone, Skin-fascia MTH, HALLUX | N | U | U | U | U | L | U | U | L |
| **Gooding 1986** | TSD, MTH | N | U | U | U | U | U | U | L | L |
| **Abouaesha 2001** | TSD, MTH | N | U | L | L | U | L | L | L | L |
| **Young 1995** | TSD, MTH | N | U | U | L | U | U | L | L | L |
| INTRINSIC FOOT MUSCLE | | | | | | | | | | |
| **Kumar 2015** | EDB, 1L, 1I, AH | N | U | U | L | U | L | U | U | L |
| **Wang 2014** | EDB, MIL | N | U | U | L | U | L | U | L | L |
| **Severinsen 2007** | EDB, MIL | N | U | U | U | U | L | L | L | L |

FPT = fat pad thickness, TSD = Total soft tissue depth, MTH = Metatarsal head, EDB = Extensor digitorum brevis muscle, MIL= *combined thickness 1st Dorsal Interosseous + Adductor hallucis + 1st Lumbrical muscles.* 1L = 1^st^ Lumbrical muscle, 1I = 1^st^ Dorsal interosseous muscle, AH = adductor halluces muscle, Y= Yes, N= No, L= Low overall risk of bias that is unlikely to significantly impact the results, U= Unclear risk of bias with potential to alter results, S= significant risk of bias resulting in reduced confidence in results.
