# Supplementary material for: Can ultrasound measures of intrinsic foot muscles and plantar soft tissues predict future diabetes-related foot disease? A systematic review
Source: PLoS One. 2018 Jun 15;13(6):e0199055. doi: 10.1371/journal.pone.0199055 (PMC6003689; doi:10.1371/journal.pone.0199055)
Supplement: S4 Table — (DOCX) [file pone.0199055.s008.docx]

**S4 Table**

| Skin Thickness | | | | | | |
| --- | --- | --- | --- | --- | --- | --- |
|  | **Method of Measurement Defined** | **Measurement Plane** | **Landmarks used for reproducibility** | **Participant positioning** | **Transducer Frequency** |  |
| Kumar 2015 | Not Defined – Image provided | Not specifically stated but images provided are in longitudinal. | Technique not explicit | Supine, Ankle Neutral | 8-15MHz |  |
| Petrofsky  2008 | Not Defined – Provided image not specific to foot | None Provided | None Provided | None Provided | 10MHz |  |
| Duffin 2002 | Not defined | The transducer was placed over head of 1^st^” metatarsal. | Technique not explicit | Prone | 7MHz |  |
| Heel Pad Thickness | | | | | | |
|  | **Method of Measurement Defined** | **Measurement Plane** | **Landmarks used for reproducibility** | **Participant positioning** | **Transducer Frequency** | **Fixation Device used** |
| Chatzistergos 2014 | Minimum bone to probe distance. | Transverse | Apex of calcaneus | Not stated (possibly prone) | 13MHz | Foot rigidly fixed in apparatus |
| Hsu CC 2009 | Soft tissue thickness between the skin and the calcaneus via summation of two measures: (i) microchamber layer and (ii) macrochamber layer. | Longitudinal | The transducer was positioned along a line connecting the second toe ray and the mid-heel. | Sitting | 10MHz | None used |
| Thomas 2003 | (i) Skin-bone thickness, (ii) skin to fascia thickness, then ratio used to compare to other outcome measures (Shore Index, Pressure). | Not Explicitly stated | Limited discussion; skin and point of maximum convexity of calcaneum. Also skin and “fascia” interface. Foot divided into 10 sections. | Supine | 7.5MHz | None Used |
| Tong 2003 | Measured the shortest distance between the calcaneal tuberosity and the skin surface. | Longitudinal | A line bisecting the participant’s foot from the plantar aspect of the 1st metatarsal head to the midpoint of the calcaneum. | Supine | 5-12MHz | Foot fixed in apparatus |
| Hsu TC 2000 | Between the skin and calcaneus | Longitudinal | The transducer was positioned along a line connecting the second toe and the mid-heel. | Prone | 10MHz | Foot fixed in apparatus |
| Gooding 1986 | Outer skin surface to soft tissue-bone interface, vertical. | Transverse | Mid heel | Prone | 10MHz | None used |
| Gooding 1985 | Not stated – Inferred skin surface to the calcaneal- soft tissue junction. | Transverse | Mid heel | Prone | 10MHz | None used |
| Sub-Metatarsal Head (MTH) Thickness | | | | | | |
|  | **Method of Measurement Defined** | **Measurement Plane** | **Landmarks used for reproducibility** | **Participant positioning** | **Transducer Frequency** | **Fixation Device used** |
| UNLOADED | | | | | | |
| Kumar 2015 | Not Specified | Longitudinal (assumed from image provided) | MTH (technique not explicit) | Supine, Ankle Neutral | 8-15MHz | None used |
| Petrofsky 2008 | None Provided | None Provided | None Provided | None Provided | 10MHz | Not stated |
| Hsu CC 2007 | M-mode (1D) images used to measure thickness rather than 2D B-Mode. | Longitudinal | Transducer aligned along each ray, but no explicit MTH positioning details provided. | Supine, Ankle Neutral | 5-12MHz | Foot fixed into apparatus |
| Thomas 2003 | (i) Skin-bone thickness, (ii) skin to fascia thickness, then ratio used to compare. | Not explicitly stated | Limited discussion; point of maximum convexity of metatarsal head. Also “fascia” interface. Foot divided into 10 sections. | Supine | 7.5MHz | None Used |
| Gooding 1986 | skin surface to soft tissue-bone interface, vertical. | Transverse | MTH (technique not explicit) | Prone | 10MHz | None used |
| LOADED | | | | | | |
| Abouaesha 2001 | Distance between the most prominent part of MTH and the skin | Longitudinal | MTH (technique not explicit) | Weight bearing (Planscan). | 3.75MHz | Planscan platform |
| Young 1995 | Skin to MTH | Longitudinal | MTH (technique not explicit) | Weight bearing (Planscan). | Not Provided | Planscan platform |

MTH = Metatarsal head, 1D= one-dimensional, 2D= two-dimensional.
